# Palliative sedation in amyotrophic lateral sclerosis: results of a nationwide survey among neurologists and palliative care practitioners in Germany

**DOI:** 10.1186/s12883-022-02681-7

**Published:** 2022-04-30

**Authors:** Laura Salzmann, Bernd Alt-Epping, Alfred Simon

**Affiliations:** 1 Department of Hematology, Oncology and Palliative Medicine, Medical Center Bad Hersfeld, Bad Hersfeld, Germany; 2 Department of Palliative Medicine, University Hospital Heidelberg, Heidelberg, Germany; 3 Academy of Ethics in Medicine, University Medical Center Goettingen, Goettingen, Germany

**Keywords:** Palliative sedation, Amyotrophic lateral sclerosis (ALS), Palliative care

## Abstract

**Background:**

Palliative sedation has become widely accepted as a method to alleviate refractory symptoms in terminally ill patients. Controversies regarding this topic especially concern the use of palliative sedation for psychological symptoms, the use in patients who are not imminently dying and the simultaneous withdrawal of life-sustaining measures. Amyotrophic lateral sclerosis (ALS) is characterized by symptoms including muscle weakness, dysphagia, dysarthria, muscle spasms and progressive respiratory insufficiency. Due to these characteristic symptoms, palliative sedation might be considered to be necessary to alleviate refractory suffering in ALS patients. However, palliative sedation in ALS is only rarely discussed in current medical literature and guidelines.

**Methods:**

A questionnaire survey was conducted among neurologists and palliative care practitioners in Germany. The participants were asked to evaluate the use of palliative sedation in different situations.

**Results:**

Two hundred and ninety-six completed questionnaires were analyzed. The results suggest high levels of support for the use of palliative sedation in ALS patients. 42% of the participants stated that they had already used palliative sedation in the treatment of ALS patients. Acceptance of palliative sedation was higher in case of physical symptoms than in case of psychological symptoms. Refusal of artificial nutrition did not lead to a lower acceptance of palliative sedation. Doctors with specialist training in palliative care had already used palliative sedation in ALS patients more often and they were more likely to accept palliative sedation in different situations than the participants without a background in palliative care.

**Conclusion:**

Our survey showed that palliative sedation in ALS is widely accepted by the attending doctors. In case of psychological symptoms, palliative sedation is looked at with more concern than in case of physical symptoms. The refusal of artificial nutrition does not result in a decreased acceptance of palliative sedation. Doctors with specialist training in palliative care are more likely to approve of palliative sedation in ALS.

**Supplementary Information:**

The online version contains supplementary material available at 10.1186/s12883-022-02681-7.

## Background

The term palliative sedation describes the use of sedating drugs (e.g. benzodiazepines or neuroleptics) to alleviate refractory symptoms in terminally ill patients [1]. Depth and duration of palliative sedation can vary, depending on the indication and aims of the therapy. On the one hand, intermittent or mild sedation can be used to temporarily shield a patient from distressing symptoms and it allows for communication with the patient and for a possible reevaluation of the situation. Deep and continuous sedation on the other hand is typically used provide symptom relief until death [2]. Although we decided to use the term ‘palliative sedation’ to describe all forms of sedation that match the above mentioned definition, the discussion about a consistent terminology is still ongoing [3].

Although palliative sedation is widely accepted by doctors and medical ethicists as a means to alleviate refractory symptoms [4–6], some aspects are still controversially discussed. In general, palliative sedation can be used to relieve physical symptoms (e.g. pain, dyspnea), psychological symptoms (e.g. panic, anxiety, refractory depression) and existential distress [1, 2, 7]. However, it is not always possible to distinguish between physical, psychological and existential distress, because they can occur at the same time and are mutually dependent. [8]. And in the case of psychological symptoms, palliative sedation is evaluated more controversially, because the course of disease can be less predictable and because severe psychological symptoms are not necessarily limited to the final stages of life-threatening diseases [1, 2, 9].

The use of palliative sedation in patients who are not imminently dying is also often controversially discussed. The European Association for Palliative Care recommends that deep continuous sedation should only be used in patients with a life expectancy of hours or days [1]. This *imminence condition* [10] is rooted in the belief that palliative sedation must not hasten death in patients who would otherwise have survived for months or years. In the case of ALS, patients may experience existential suffering and/or refractory psychological symptoms like panic and depression due to the anticipated course of disease [8]. If a patient therefore requests palliative sedation, doctors face difficult decisions and the question remains how to treat insufferable and refractory symptoms in patients who are not imminently dying, and how to monitor sedation in a situation that is not close to death but nevertheless incurable and burdensome [5, 8, 11].

Furthermore, the withdrawal of life-sustaining measures like artificial nutrition or mechanical ventilation under palliative sedation is controversially discussed among medical ethicists. Some authors express the belief that this course of action can be classified as euthanasia, because it intentionally shortens a patient’s life [12]. Others argue that every person has a right to refuse life-sustaining measures and that this must not affect the indication for palliative sedation [8]. According to German law, the withdrawal of life-sustaining measures under palliative sedation is legal, if the treatment is discontinued in accordance with the wishes of the patient and the sedation is medically indicated to alleviate stressful situations during the dying phase. An unintended shortening of life as a side effect of the sedation may be accepted [13].

As a motoneuron disease, amyotrophic lateral sclerosis (ALS) results in a loss of voluntary motor control. Characteristic symptoms are muscle weakness, dysphagia, dysarthria, muscle atrophy, muscle spasms, respiratory insufficiency and dyspnea [14]. In most cases, ALS progresses rapidly, with an average life expectancy at diagnosis of two to four years [15]. As ALS cannot be cured, treatment includes the neuroprotective drug Riluzole, a benzothiazole derivative which may prolong survival by several months, invasive and non-invasive ventilation, supportive therapy, such as physical therapy, occupational therapy, the prevention of infections, and analgesia and palliative care [14–16]. The antioxidant Edaravone is approved in the USA and in Japan for the treatment of ALS, but not in Europe [17].

Palliative sedation is most often discussed in the context of oncological patients, because these form the majority of patients receiving palliative care services [18]. Due to the characteristic symptoms (e.g. dyspnea, dysphagia, muscle spasms, muscle weakness) that are associated with ALS and due to the lack of a curative therapy it seems reasonable to include palliative care and therefore also palliative sedation as therapeutic options [13, 19]. The German Society for Neurology emphasizes the importance of palliative care in ALS treatment, whereas palliative sedation is not explicitly mentioned [15].

The aim of our study was to investigate whether palliative sedation is used in the treatment of ALS patients in Germany and how the attending doctors evaluate the use of palliative sedation in ALS. We wanted to find out how the attending doctors assess the use of palliative sedation if an ALS patient suffers from physical symptoms on the one hand and from psychological symptoms on the other. We also wanted to know how a patient’s refusal of artificial nutrition affects the physicians’ approval of palliative sedation. We were furthermore interested in how the attending doctors think about the withdrawal of artificial ventilation under palliative sedation in an ALS patient. Lastly, we wanted to know how a specialist training in palliative care might have affected the physicians’ attitude towards palliative sedation in ALS patients.

### Methods

A questionnaire survey was conducted among German neurologists – who are most likely to treat ALS patients – and palliative care practitioners – who are most likely to perform palliative sedation – in Germany. The greater part of the questionnaire consisted of five case examples. In case example 1.1, an ALS patient expresses her wish for deep and continuous palliative sedation due to intolerable physical symptoms (dyspnea, dysphagia, dysarthria, pain). In case example 1.2, the same patient additionally refuses artificial nutrition during palliative sedation. In case examples 2.1 and 2.2, a similar scenario is described but now the ALS patient suffers from intolerable psychological symptoms (fear, panic, depression, existential suffering). In case example 3, an artificially ventilated ALS patients asks for deep and continuous palliative sedation and a simultaneous withdrawal of mechanical ventilation. Further questions address the participants’ experience with palliative sedation and ALS, their specialist medical training and sociodemographic data (age, gender).

The survey was conducted in May 2018, 570 neurologists and 423 palliative care practitioners were contacted. The contact information of all registered neurologists and palliative care practitioners (1794 doctors) was taken from the websites of the State Medical Boards and a random sample of 993 doctors was contacted.

The data were analyzed using Statistica 13.3 and SPSS 25.0. The Mann–Whitney U test was used to compare two independent groups when the dependent variable was ordinal. The Kruskal-Wallis test was used to compare more than two independent groups when the dependent variable was ordinal. For a comparison of two related groups and an ordinal dependent variable, the Wilcoxon signed-rank test was used. The Chi-Square test was used to analyze nominal variables. The significance level was set to 5% and the Bonferroni correction was used to adjust the significance level in case of multiple testings.

## Results

At a response rate of 30%, 296 completed questionnaires were statistically analyzed. At the time of the survey, 245 respondents (85%) were 45 years or older and 203 (79%) were male. As to the respondents’ medical specialization, neurology was named by 129 participants (44%), internal medicine by 78 (27%) and anesthesiology by 53 (18%). 182 participants (63%) had completed specialist training in palliative medicine (Table 1).

**Table 1:** Socio-demographic data

Age (years)	*N* = 289
< 35	*N* = 0 (0%)
35–45	*N* = 44 (15, 2%)
45–55	*N* = 119 (41, 2%)
55–65	*N* = 115 (39, 8%)
> 65	*N* = 11 (3, 8%)
**Gender**	***N* = 291**
Female	*N* = 87 (29, 9%)
Male	*N* = 203 (69, 8%)
Other / n.s	*N* = 1 (0, 3%)
**Medical specialization**	***N* = 291**
Neurology	*N* = 129 (44, 3%)
Anesthesiology	*N* = 53 (18, 2%)
Internal medicine	*N* = 78 (26, 8%)
Other	*N* = 61 (21%)
**Specialist training in palliative care**	***N* = 291**
Yes	*N* = 182 (62, 8%)
No	*N* = 108 (37, 2%)

Overall, 194 participants (66%) stated that they had already dealt intensively with the issue of palliative sedation. Among the respondents with specialist training in palliative care, this number was significantly higher (94%) (Table 2). 225 participants (77%) indicated that they treated fewer than three ALS patients monthly on average, 49 (17%) treated no ALS patients at all (Table 3). Overall, 123 (41,8%) had already prescribed palliative sedation in patients with ALS. The participants with specialist training in palliative care had performed palliative sedation in ALS patients significantly more often (52% vs. 26%) (Table 4). As reasons for the use of palliative sedation, dyspnea and fear were mentioned most often (by 95 participants).

**Table 2:** Are you familiar with the term ‘palliative sedation’?

	**Overall (*N* = 294)**	**Specialist training in palliative care (*N* = 182)**	**Ø Specialist training in palliative care (*N* = 107)**
**I have never heard of the term.**	*N* = 16 (5,4%)	*N* = 0 (0%)	*N* = 16 (14,9%)
**I have heard of the term, but I have not yet dealt with the topic.**	*N* = 27 (9,2%)	*N* = 1 (0,5%)	*N* = 26 (24,3%)
**I have heard of the term and I have dealt with the topic a bit.**	*N* = 57 (19,4%)	N = 10 (5,5%)	*N* = 46 (43%)
**I have heard of the topic and I have dealt with the topic intensively.**	*N* = 194 (66%)	*N* = 171 (94%)	*N* = 19 (17,8%)

**Table 3:** How many ALS patients do you treat per month?

	**Overall *N* = 291**	**Specialist training in palliative care *N* = 179**	**Ø Specialist training in palliative care N = 107**
**None**	*N* = 49 (16, 8%)	*N* = 37 (20, 6%)	*N* = 9 (8, 4%)
** < 3**	*N* = 225 (77, 3%)	*N* = 136 (76%)	*N* = 88 (82, 2%)
** 4–10**	*N* = 13 (4, 5%)	*N* = 5 (2, 8%)	*N* = 8 (7, 5%)
** > 10**	*N* = 4 (1, 4%)	*N* = 1 (0, 6%)	*N* = 2 (1, 9%)

**Table 4:** Have you already performed palliative sedation in ALS patients?

	**Overall *N* = 294**	**Specialist training in palliative care *N* = 182**	**Ø Specialist training in palliative care *N* = 107**
** Yes**	*N* = 123 (41, 8%)	*N* = 95 (52, 2%)	*N* = 28 (26, 2%)
** No**	*N* = 171 (58, 2%)	*N* = 87 (47, 8%)	*N* = 79 (73,8%)

### Palliative sedation in an ALS patient with intolerable physical symptoms

In case example 1.1, an ALS patient expresses the wish for palliative sedation due to intolerable physical symptoms. Almost all the participants (99%) could relate to the patient’s wish and 247 (87%) thought that the patient has a right to be sedated. 241 (85%) would only use deep and continuous palliative sedation if superficial or intermittent sedation had proven unsuccessful. 66 respondents (23%) would limit the use of palliative sedation to imminently dying patients and 13 (5%) would not use palliative sedation at all in this case.

### Refusal of artificial nutrition under palliative sedation in an ALS patient with intolerable physical symptoms

In case example 1.2, 283 respondents (98%) could relate to the patient’s wish for palliative sedation and a simultaneous refusal of artificial nutrition. 255 (90%) stated that the patient has a right to be sedated without artificial nutrition. 82 (71%) would meet the patient’s wish even if it was not documented in an advance directive and 229 participants (80%) would not restrict palliative sedation without artificial nutrition to imminently dying patients. Also, 265 (93%) did not classify palliative sedation without artificial nutrition as euthanasia. 160 (57%) believed that palliative sedation without artificial nutrition would hasten death in this case and 211 (76%) expressed the view that artificial nutrition is not indicated during a deep continuous sedation.

### Palliative sedation in an ALS patient with intolerable psychological symptoms

In case example 2.1 an ALS patient requests palliative sedation due to intolerable psychological symptoms. 270 respondents (93%) could understand the patient’s wish and 195 (70%) believed that the patient has a right to palliative sedation. 232 (84%) would only agree to a deep continuous sedation if a superficial or intermittent sedation had proven unsuccessful. In this case, 94 (33%) would limit palliative sedation to imminently dying patients and 21 (7%) would not use palliative sedation at all.

### Refusal of artificial nutrition under palliative sedation in an ALS patient with intolerable psychological symptoms

In case example 2.2, 275 participants (96%) stated that they could understand the patient’s wish and 219 (78%) believed that she had a right to be sedated without artificial nutrition. 186 (67%) would meet the patient’s wish even if it was not documented in an advance directive and in this case 193 (68%) refused to limit palliative sedation without artificial nutrition to imminently dying patients. 256 (90%) did not classify palliative sedation without artificial nutrition as euthanasia in this case. 176 (64%) thought that palliative sedation without artificial nutrition would hasten death in this case and 187 (70%) stated that artificial nutrition is not indicated during a deep continuous sedation.

### Withdrawal of artificial ventilation under palliative sedation

In case example 3, an artificially ventilated ALS patient expresses the wish for a withdrawal of ventilation under palliative sedation. All participants (100%) stated that they could relate to the patient’s wish and 270 (95%) believed that she has a right to discontinue mechanical ventilation under palliative sedation. 200 (70%) would meet the request even if it was not documented in an advance directive and 227 (80%) agreed to this approach even if the patient was not imminently dying. 248 (88%) respondents did not classify the withdrawal of mechanical ventilation under palliative sedation as euthanasia.

### A comparison of the participants with and without specialist training in palliative care

Overall, the participants with specialist training in palliative care responded more favorably to the use of palliative sedation. In case example 1.2 (wish for palliative sedation due to physical symptoms and refusal of artificial nutrition), they could relate to the patient’s wish more often than those without training in palliative care (*p* = 0.00001). They also regarded an advance directive as less important for the decision-making process (*p* = 0.00016) and they were less likely to restrict palliative sedation without artificial nutrition to imminently dying patients (*p* < 0.00001). Moreover, they less frequently classified the requested course of action as euthanasia (*p* < 0.00001) and they were less likely to believe that it would hasten death (*p* = 0.00019). Also, they more often expressed the opinion that artificial nutrition is not indicated during a deep continuous sedation (*p* = 0.00008).

In case example 2.1 (wish for palliative sedation due to psychological symptoms) the participants with specialist training in palliative care could again relate to the patient’s wish more often (*p* = 0.00004) and were less likely to restrict palliative sedation to imminently dying patients (*p* = 0.00116). Also, they less frequently stated that they would not perform palliative sedation at all in this case (*p* = 0.00005).

In case example 2.2 (wish for palliative sedation due to psychological symptoms and refusal of artificial nutrition) the respondents with specialist training in palliative care could relate more often to the patient’s wish (*p* < 0.00001), they more often believed that the patient has a right to the requested course of action (*p* = 0.00029) and they once more attached less importance to an advance directive (*p* = 0.00084). Again, they were less likely to restrict palliative sedation without artificial nutrition to imminently dying patients in this case (*p* = 0.00001) and they less frequently classified the requested course of action as euthanasia (*p* < 0.00001). Also, they stated less often that the patient’s death would be hastened (*p* = 0.00007) and they more frequently expressed the view that in this case, artificial nutrition is not indicated during continuous deep sedation (*p* = 0.00005).

In case example 3 (wish for withdrawal of mechanical ventilation and palliative sedation), the participants with specialist training in palliative care did again attach less importance to an advance directive (*p* = 0.00129), they were less likely to restrict the requested course of action to imminently dying patients (*p* < 0.00001) and they less frequently classified the withdrawal of mechanical ventilation under palliative sedation as euthanasia (*p* < 0.00001).

### A comparison of the five case examples (Figs. 1, 2 and 3)

**Fig. 1:**
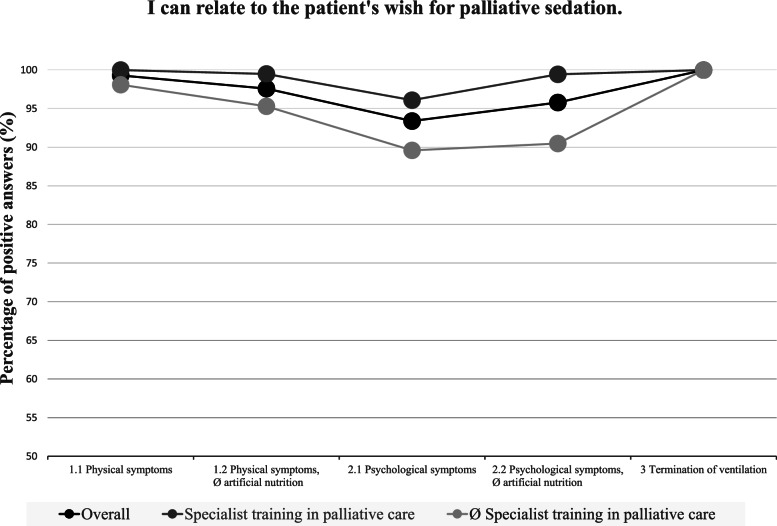
Positive answers to “I can relate to the patient's wish for palliative sedation”

**Fig. 2:**
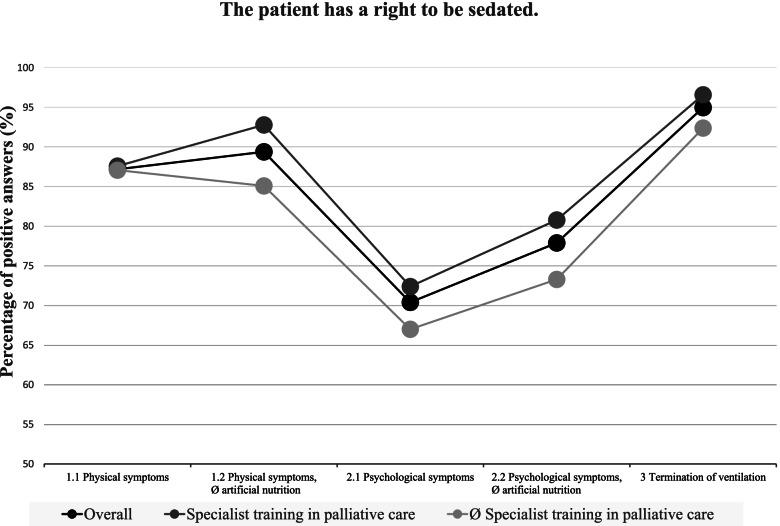
Positive answers to “The patient hast a right to be sedated”

**Fig. 3:**
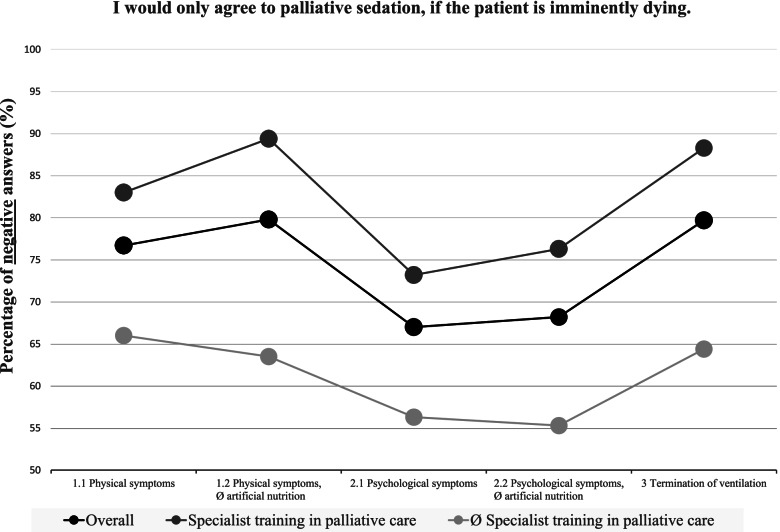
Negative answers to “I would only agree to palliative sedation, if the patient is imminently dying”

The comparison of the five case examples revealed that palliative sedation in patients with physical symptoms is, on the whole, more widely accepted than palliative sedation in patients with psychological symptoms. As illustrated in Figs. 1, 2 and 3, in case example 1.1 (wish for palliative sedation due to physical symptoms), the participants could relate more often to the patient’s wish for palliative sedation (*p* < 0.00001), they were more likely to believe that the patient has a right to be sedated (*p* < 0.00001) and they would less often restrict the requested actions to an imminently dying patient (*p* = 0.00004) than in case example 2.1 (wish for palliative sedation due to psychological symptoms).

Similar results can be seen when comparing case examples 1.2 (wish for palliative sedation due to physical symptoms and refusal of artificial nutrition) and 2.2 (wish for palliative sedation due to psychological symptoms and refusal of artificial nutrition). Again, in the case of physical symptoms, the participants were more likely to believe that the patient has a right to the requested course of action (*p* < 0.00001) and they would less often restrict this course of action to imminently dying patients (*p* < 0.00001).

The refusal of artificial nutrition however did not result in a decreased acceptance of palliative sedation. On the contrary, in case example 1.2 (wish for palliative sedation due to physical symptoms and refusal of artificial nutrition), the participants more often believed the patient to have a right to the requested course of action (*p* = 0.00006) than in case example 1.1 (wish for palliative sedation due to physical symptoms).

This tendency also becomes apparent when comparing case examples 2.1 (wish for palliative sedation due to psychological symptoms) and 2.2 (wish for palliative sedation due to psychological symptoms and refusal of artificial nutrition). In the case of a refusal of artificial nutrition, the respondents more often stated that the patient has a right to the requested course of action (*p* < 0.00001).

Overall, the participants reacted very approvingly to the provision of simultaneous palliative sedation when a person with ALS requested the withdrawal of mechanical ventilation (case example 3). In case example 3, they could relate to the patients wish more often than in case examples 1.1 and 2.1 (*p* < 0.00001), they more often believed that the patient has a right to his wishes than in case examples 1.1 and 2.1 (*p* < 0.00001) and they were less likely to limit the requested course of action to an imminently dying patient than in case example 2.1 (*p* < 0.00001).

## Discussion

Overall, our survey revealed high acceptance ratings of palliative sedation among the participating doctors. Acceptance of palliative sedation was higher when patients with physical rather than psychological symptoms were concerned. A majority would not limit the use of palliative sedation to imminently dying patients and a patient’s refusal of artificial nutrition did not lead to a lower acceptance of palliative sedation. Also, the wish for a termination of mechanical ventilation and a simultaneous palliative sedation was accepted by most participants. Our findings also showed that those doctors with specialist training in palliative care tend to react more favorably towards palliative sedation in various contexts.

### Palliative sedation as part of the treatment of ALS patients

Forty-two percent of all respondents stated that they had already used palliative sedation to treat ALS patients. Among the participating doctors with specialist training in palliative care, the percentage was significantly higher (52,2%) than among those without this background in palliative care (26,2%). As indications, dyspnea and fear were named most frequently. Considering that palliative sedation in ALS is only rarely discussed in the current neurological literature, these results were unexpected.

### Palliative sedation as a means to alleviate physical and psychological symptoms in ALS patients

Overall, palliative sedation was judged an adequate method to treat refractory symptoms in ALS patients by the participating doctors. However, in the context of psychological symptoms, the acceptance ratings were significantly lower than in the context of physical symptoms. Less than 5% indicated that they would not use palliative sedation at all to alleviate physical symptoms, compared with 7% in the case of psychological symptoms. The participants were also more likely to understand the wish for palliative sedation and to grant the patient the right to receive palliative sedation in the case of physical symptoms and they were less likely to limit palliative sedation to imminently dying patients. These results were not unexpected, because the use of palliative sedation for psychological symptoms is controversially discussed in medical ethical literature, as it is more difficult to evaluate if psychological symptoms are persistent [1, 2].

Still, even in the case of psychological symptoms, the majority of the participants could relate to the ALS patient’s wish and 70% stated that the patient has a right to be sedated. These numbers differ from the results of a survey that was conducted among members of the German Academy for Ethics in Medicine in 2007 [20]. In the context of this survey, the use of palliative sedation to alleviate physical symptoms in a dying patient was deemed morally acceptable by over 97%, compared with around 60% in case of psychological symptoms. Of course, the two surveys are only partially comparable, but it seems possible that the attitude towards palliative sedation for psychological symptoms has changed over the last years.

With regard to the timing of palliative sedation, 23% of the participants would limit palliative sedation to imminently dying ALS patients (with physical symptoms). In the context of a survey by Russel et al. (2010) that was conducted among American neurologists, 92% of the participating doctors would accept sedation for cancer patients who are imminently dying, while only 50% agreed that sedation was acceptable for end-stage ALS patients [21].

### Palliative sedation and the refusal of artificial nutrition

The refusal of artificial nutrition under palliative sedation is critically discussed among medical ethicists, as this course of action has the potential to significantly shorten a patient’s life [22]. Some authors have even classified this method as euthanasia in the past [12]. Others point out that the decision about palliative sedation and the decision about life-supporting measures must be made separately, because every patient has the right to refuse life-supporting measures and this decision does not influence the indication for palliative sedation [8, 23]. The results of our survey are surprising, as the refusal of artificial nutrition did not lead to a lower acceptance of palliative sedation among the participating doctors. On the contrary, in the case of a refusal of artificial nutrition, the respondents were more likely to think that the ALS patients has a right to be sedated.

Also, over 90% of the participants refused to classify this course of action as euthanasia. In the light of earlier surveys, these results were unexpected. For example, a survey by van Oorschot et al. [24] suggested that 25% of the participating doctors classified the withdrawal of artificial hydration (independent of palliative sedation) as euthanasia. However, it is possible that the term “killing on request” that was used in our questionnaire was defined in different ways by the participants. An uncertainty about the term has already been described in former surveys [24, 25] and the topic has also been addressed by some of our participants.

### Palliative sedation and the withdrawal of mechanical ventilation

The withdrawal of mechanical ventilation in ALS patients has to be managed carefully, both because it involves a life-ending decision on the part of the patient and because ALS patients are likely to develop severe dyspnea during the withdrawal process [26]. However, with regards to both medical-ethical guidelines and to current legislation, every patient has the right to decide against the continuation of mechanical ventilation [26–28].

The participating doctors reacted approvingly towards the described patient’s wish for palliative sedation and the simultaneous withdrawal of mechanical ventilation. 95% indicated that the patient has a right to these measures. These high acceptance ratings were to a certain extent unexpected, as some surveys suggest an uncertainty about the legitimacy of the withdrawal of mechanical ventilation among doctors [24, 25]. However, more recent surveys indicate a more accepting attitude towards the withdrawal of mechanical ventilation [29].

From a medical ethical point of view and also with regards to current jurisdiction, these findings are to be welcomed, as every patient must be able to decide against the continuation of mechanical ventilation and this process usually has to be accompanied by palliative sedation, in order to prevent distressing symptoms [26, 30].

### The physicians’ education and training influence their attitude towards palliative sedation

Analyzing the participants’ responses, a significant difference between those doctors with specialist training in palliative medicine and those without became obvious. On the one hand, 52% of the respondents with specialist training in palliative medicine had already performed palliative sedation in an ALS patient, compared with 26% of the respondents without this background in palliative medicine. On the other hand, the participants without specialist training in palliative care had significantly more experience with the treatment of ALS patients.

Moreover, the participants with specialist training in palliative care reacted more approvingly towards the use of palliative sedation in ALS. They were less likely to reject an ALS patient’s wish for palliative sedation and to limit palliative sedation to imminently dying patients. They also attached less importance to an advance directive, they were more likely to accept the simultaneous refusal of artificial nutrition and they less frequently classified this course of action as euthanasia than the respondents without specialist training in palliative care. The doctors with specialist training in palliative care also reacted more positively towards the use of palliative sedation for psychological symptoms and they more often stated that artificial nutrition is not indicated during continuous and deep sedation.

It is plausible that those doctors who have dealt with the topic of palliative sedation during their training react more approvingly towards the use of palliative sedation in ALS. Also, their attitudes towards palliative sedation more often comply with the current medical ethical discussion of the topic and with the current jurisdiction.

## Conclusions

Our survey showed that palliative sedation in ALS is widely accepted among attending neurologists and palliative care specialists in Germany. The acceptance of palliative sedation is significantly higher when physical symptoms rather than psychological symptoms are concerned. An ALS patient’s refusal of artificial nutrition did not lead to a decreased acceptance of palliative sedation. Also, the withdrawal of mechanical ventilation under palliative sedation was accepted by the majority of the participants. Overall, the participants with specialist training in palliative care reacted more approvingly towards the use of palliative sedation in ALS.

From a medical ethical point of view, the fact that non-oncological patients have access to palliative care and palliative sedation must be appreciated. Our results emphasize how important it is to include therapeutic concepts of palliative care as well as palliative care specialists into the treatment of ALS patients, in order to achieve profound symptom control even in refractory cases.

## Supplementary Information

**Additional file 1.**

**Additional file 2.**

## Data Availability

All data generated or analyzed during this study are included in this published article and its supplementary information files.

## Abbreviation

ALS: Amyotrophic lateral sclerosis
