# Correction: Palliative sedation in amyotrophic lateral sclerosis: results of a nationwide survey among neurologists and palliative care practitioners in Germany

**DOI:** 10.1186/s12883-022-02718-x

**Published:** 2022-05-23

**Authors:** Laura Salzmann, Bernd Alt-Epping, Alfred Simon

**Affiliations:** 1 Department of Hematology, Oncology and Palliative Medicine, Medical Center Bad Hersfeld, Bad Hersfeld, Germany; 2 Department of Palliative Medicine, University Hospital Heidelberg, Heidelberg, Germany; 3 Academy of Ethics in Medicine, University Medical Center Goettingen, Goettingen, Germany

**Correction: BMC Neurol 22, 161 (2022)**

**https://doi.org/10.1186/s12883-022-02681-7**

Following publication of the original article [1], an error was reported in the reference list wherein reference 3 is not correct.

Incorrect:

Intentional sedation as a means to ease suffering: a systematically constructed terminology for sedation in palliative care. https://www.liebertpub.com/doi/epdf/https://doi.org/10.1089/jpm.2021. 0428. Accessed 13 Mar 2022.

Correct:

Kremling, A.; Bausewein, C.; Klein, C.; Schildmann, E.; Ostgathe, C.; Ziegler, K. & Schildmann, J. Intentional Sedation as a Means to Ease Suffering: A Systematically Constructed Terminology for Sedation in Palliative Care, J Palliat Med, 2022;25:5

The original article [1] has been updated.
