# Supplementary material for: Palliative sedation in amyotrophic lateral sclerosis: results of a nationwide survey among neurologists and palliative care practitioners in Germany
Source: BMC Neurol. 2022 Apr 30;22:161. doi: 10.1186/s12883-022-02681-7 (PMC9055769; doi:10.1186/s12883-022-02681-7)
Supplement: Supplementary file 1 — Additional file 1. [file 12883_2022_2681_MOESM1_ESM.pdf]

Akademie für Ethik in der Medizin — A. Simon, B. Alt-Epping, L.Salzmann

Universitätsmedizin Göttingen — Palliative Sedierung bei ALS

Bitte so markieren: ☐ ☒ ☐ ☐ ☐ Bitte verwenden Sie einen Kugelschreiber oder nicht zu starken Filzstift. Dieser Fragebogen wird maschinell erfasst.

Korrektur: ☐ ■ ☐ ☒ ☐ Bitte beachten Sie im Interesse einer optimalen Datenerfassung die links gegebenen Hinweise beim Ausfüllen.

Der folgende Fragebogen thematisiert den Einsatz einer Palliativen Sedierung in der Behandlung von ALS-Patientinnen und ALS-Patienten.

Das Handlungskonzept der Palliativen Sedierung ist definiert als „der überwachte Einsatz von Medikamenten mit dem Ziel einer verminderten oder aufgehobenen Bewusstseinslage (Bewusstlosigkeit), um die Symptomlast in anderweitig therapierefraktären Situationen in einer für Patienten, Angehörigen und Mitarbeitern ethisch akzeptablen Weise zu reduzieren“ (European Association for Palliative Care 2010).

Bitte schicken Sie den ausgefüllten Fragebogen <u>bis zum 24.06.2018</u> zusammen mit dem Deckblatt an die auf dem Deckblatt angegebene Adresse.

Sind Sie mit dem Begriff der Palliativen Sedierung vertraut?

☐ Ich habe den Begriff bisher noch nie gehört.
☐ Mir ist der Begriff bekannt, ich habe mich mit dem Thema jedoch noch nicht beschäftigt.
☐ Mir ist der Begriff bekannt und ich habe mich bereits ein wenig mit dem Thema beschäftigt.
☐ Mir ist der Begriff bekannt und ich habe mich bereits intensiv mit dem Thema beschäftigt.

Wie viele ALS-Patientinnen und ALS-Patienten behandeln Sie im Durchschnitt pro Monat?

☐ keine
☐ unter 3
☐ 4 - 10
☐ über 10

Wie lange behandeln Sie schon ALS-Patientinnen und ALS-Patienten?

☐ Bisher keine Erfahrung
☐ unter 5 Jahre
☐ 5 - 10 Jahre
☐ 10- 15 Jahre
☐ über 15 Jahre

Haben Sie bereits eine Palliative Sedierung bei einer ALS-Patientin bzw. einem ALS-Patienten durchgeführt?

☐ Ja
☐ Nein

Wenn ja, bei welchen therapierefraktären Symptomen?

Fallbeispiel 1

Sie behandeln eine ALS-Patientin im fortgeschrittenen Krankheitsstadium. Die Patientin hat starke körperliche Symptome (Dyspnoe, Pseudohypersalivation, Dysphagie, Dysarthrie, Schmerzen durch Spastik und Muskelkrämpfe), die bisher nicht in ausreichendem Maße gelindert werden konnten. Die Patientin ist dadurch offensichtlich stark belastet.

Die Patientin ist einwilligungsfähig und wünscht aufgrund der refraktären körperlichen Symptomatik eine kontinuierliche und tiefe Sedierung.
Bitte geben Sie für jede Aussage an, inwieweit Sie dieser zustimmen.

	Stimme völlig zu	Stimme eher zu	Stimme eher nicht zu	Stimme nicht zu	Keine Angabe
Der Wunsch der Patientin ist für mich nachvollziehbar.	☐	☐	☐	☐	☐
Die Patientin hat ein Recht darauf, dass ihrem Wunsch entsprochen wird.	☐	☐	☐	☐	☐
Ich würde dem Wunsch nur nachkommen, wenn vorherige Versuche einer flachen bzw. intermittierenden Sedierung keine ausreichende Symptomlinderung erzielen konnten.	☐	☐	☐	☐	☐
Ich würde dem Wunsch nur nachkommen, wenn sich die Patientin bereits unmittelbar in der Sterbephase befindet.	☐	☐	☐	☐	☐
Ich würde generell keine kontinuierliche tiefe Sedierung durchführen.	☐	☐	☐	☐	☐

Die Patientin ist einwilligungsfähig und wünscht aufgrund der refraktären körperlichen Symptomatik eine kontinuierliche und tiefe Sedierung. Eine künstliche Ernährung während der Sedierung lehnt sie ausdrücklich ab.
Bitte geben Sie für jede Aussage an, inwieweit Sie dieser zustimmen.

	Stimme völlig zu	Stimme eher zu	Stimme eher nicht zu	Stimme nicht zu	Keine Angabe
Der Wunsch der Patientin ist für mich nachvollziehbar.	☐	☐	☐	☐	☐
Die Patientin hat ein Recht darauf, dass ihrem Wunsch entsprochen wird.	☐	☐	☐	☐	☐
Ich würde dem Wunsch nur nachkommen, wenn dieser in einer Patientenverfügung dokumentiert ist.	☐	☐	☐	☐	☐
Ich würde dem Wunsch nur nachkommen, wenn sich die Patientin bereits unmittelbar in der Sterbephase befindet.	☐	☐	☐	☐	☐
Der Verzicht auf künstliche Ernährung bei kontinuierlicher tiefer Sedierung wäre in meinen Augen mit einer Tötung auf Verlangen gleichzusetzen.	☐	☐	☐	☐	☐
Der Verzicht auf künstliche Ernährung bei kontinuierlicher und tiefer Sedierung führt zu einem früheren Versterben als es dem Erkrankungsverlauf ohne diese Maßnahme entsprechen würde.	☐	☐	☐	☐	☐
Eine künstliche Ernährung bei einer kontinuierlichen tiefen Sedierung wäre in meinen Augen medizinisch nicht indiziert.	☐	☐	☐	☐	☐

Welches Vorgehen würden Sie im Kontext der zuletzt beschriebenen Situation als moralisch belastender empfinden?

- ☐ Abbruch einer bestehenden künstlichen Ernährung über PEG mit Beginn der Sedierung.
- ☐ Sedierung einer bisher noch selbstständig essenden und trinkenden Patientin und Verzicht auf künstliche Ernährung während der Sedierung.
- ☐ Ich würde beide Szenarien als gleich belastend empfinden.

Fallbeispiel 2

Sie behandeln eine ALS-Patientin im fortgeschrittenen Krankheitsstadium. Die Patientin weist starke psychische Symptome (Angst, Panik, Depression) und Belastungen im Sinne existenziellen Leids auf. Diese Symptomatik konnte bisher nicht in ausreichendem Maße gelindert werden. Die Patientin ist dadurch offensichtlich stark belastet.

Die Patientin ist einwilligungsfähig und wünscht aufgrund der refraktären psychischen und existenziellen Symptomatik eine kontinuierliche und tiefe Sedierung.
Bitte geben Sie für jede Aussage an, inwieweit Sie dieser zustimmen.

	Stimme völlig zu	Stimme eher zu	Stimme eher nicht zu	Stimme nicht zu	Keine Angabe
Der Wunsch der Patientin ist für mich nachvollziehbar.	☐	☐	☐	☐	☐
Die Patientin hat ein Recht darauf, dass ihrem Wunsch entsprochen wird.	☐	☐	☐	☐	☐
Ich würde dem Wunsch nur nachkommen, wenn vorherige Versuche einer flachen bzw. intermittierenden Sedierung keine ausreichende Symptomlinderung erzielen konnten.	☐	☐	☐	☐	☐
Ich würde dem Wunsch nur nachkommen, wenn sich die Patientin bereits unmittelbar in der Sterbephase befindet.	☐	☐	☐	☐	☐
Ich würde generell keine kontinuierliche tiefe Sedierung durchführen.	☐	☐	☐	☐	☐

Die Patientin ist einwilligungsfähig und wünscht aufgrund der refraktären psychischen und existenziellen Symptomatik eine kontinuierliche und tiefe Sedierung. Eine künstliche Ernährung während der Sedierung lehnt sie ausdrücklich ab.
Bitte geben Sie für jede Aussage an, inwieweit Sie dieser zustimmen.

	Stimme völlig zu	Stimme eher zu	Stimme eher nicht zu	Stimme nicht zu	Keine Angabe
Der Wunsch der Patientin ist für mich nachvollziehbar.	☐	☐	☐	☐	☐
Die Patientin hat ein Recht darauf, dass ihrem Wunsch entsprochen wird.	☐	☐	☐	☐	☐
Ich würde dem Wunsch nur nachkommen, wenn dieser in einer Patientenverfügung dokumentiert ist.	☐	☐	☐	☐	☐
Ich würde dem Wunsch nur nachkommen, wenn sich die Patientin bereits unmittelbar in der Sterbephase befindet.	☐	☐	☐	☐	☐
Der Verzicht auf künstliche Ernährung bei kontinuierlicher tiefer Sedierung wäre in meinen Augen mit einer Tötung auf Verlangen gleichzusetzen.	☐	☐	☐	☐	☐
Der Verzicht auf künstliche Ernährung bei kontinuierlicher und tiefer Sedierung führt zu einem früheren Versterben als es dem Erkrankungsverlauf ohne diese Maßnahme entsprechen würde.	☐	☐	☐	☐	☐
Eine künstliche Ernährung bei einer kontinuierlichen tiefen Sedierung wäre in meinen Augen medizinisch nicht indiziert.	☐	☐	☐	☐	☐

Welches Vorgehen würden Sie im Kontext der zuletzt beschriebenen Situation als moralisch belastender empfinden?

- ☐ Abbruch einer bestehenden künstlichen Ernährung über PEG mit Beginn der Sedierung.
- ☐ Sedierung einer bisher noch selbstständig essenden und trinkenden Patientin und Verzicht auf künstliche Ernährung während der Sedierung.
- ☐ Ich würde beide Szenarien als gleich belastend empfinden.

Fallbeispiel 3

Sie behandeln einen ALS-Patienten im Endstadium der Erkrankung, der aufgrund fortschreitender respiratorischer Insuffizienz seit einigen Monaten invasiv (über eine Trachealkanüle) beatmet wird. Der Patient weist zudem eine starke körperliche Symptomatik mit fortgeschrittener Tetraparese, Dysphagie, Dysarthrie, Schmerzen durch Spastik und Muskelkrämpfe auf und ist dadurch offensichtlich stark belastet.

Der Patient ist einwilligungsfähig und wünscht nun eine Beendigung der invasiven Beatmung bei gleichzeitiger Palliativer Sedierung.
Bitte geben Sie für jede Aussage an, inwieweit Sie dieser zustimmen.

	Stimme völlig zu	Stimme eher zu	Stimme eher nicht zu	Stimme nicht zu	Keine Angabe
Der Wunsch des Patienten ist für mich nachvollziehbar.	☐	☐	☐	☐	☐
Der Patient hat ein Recht darauf, dass seinem Wunsch entsprochen wird.	☐	☐	☐	☐	☐
Ich würde dem Wunsch nur nachkommen, wenn dieser in einer Patientenverfügung dokumentiert ist.	☐	☐	☐	☐	☐
Ich würde dem Wunsch nur nachkommen, wenn sich der Patient bereits unmittelbar in der Sterbephase befindet.	☐	☐	☐	☐	☐
Die Beendigung der invasiven Beatmung unter Sedierung wäre in meinen Augen mit einer Tötung auf Verlangen gleichzusetzen.	☐	☐	☐	☐	☐

Persönliche Daten

Geschlecht:
☐ weiblich ☐ männlich ☐ anderes / Keine Angabe

Alter:
☐ unter 35 ☐ 35 - 45 ☐ 45 - 55
☐ 55 - 65 ☐ über 65

Welche Facharztausbildung(en) haben Sie absolviert?
☐ Neurologie ☐ Anästhesiologie ☐ Innere Medizin
☐ Sonstige

Haben Sie die Zusatz-Weiterbildung Palliativmedizin absolviert?
☐ Ja ☐ Nein

Haben Sie weitere Anmerkungen zum Thema oder zur Befragung?
